# Outcome Measures in Mental Health – RCPsych Report and Working Group Survey

**DOI:** 10.1192/bjo.2024.421

**Published:** 2024-08-01

**Authors:** Jonathan Richardson, Howard Ryland, Rahul Bhattacharya

**Affiliations:** 1 Cumbria, Northumberland, Tyne and Wear NHS FT, Newcastle upon Tyne, United Kingdom; 2 Oxford Health NHS Foundation Trust, Oxford, United Kingdom; 3 East London NHS FT, London, United Kingdom

## Abstract

**Aims:**

Outcome measurement is central to transforming mental health care by quantifying change, enabling comparison and driving improvement. In recognition of this, the Royal College of Psychiatrists (RCPsych) has established a *working group on outcome measures*, led by an Associate Registrar.

To support routine outcome measurement capture in clinical services, RCPsych has developed the ‘Outcome Measurement in Psychiatry’ report.

The working group intends to launch a survey of Members to:

1. Understand psychiatrists’ current use of outcome measures.
2. Understand psychiatrists’ views on barriers and facilitators to the use of outcome measures.
3. Get feedback on the College Report.

**Methods:**

The ‘Outcome Measurement in Psychiatry’ report was developed with input from all RCPsych Faculties and is scheduled for publication prior to the RCPsych International Congress.

Feedback will be sought on the ‘Outcome Measurement in Psychiatry’ report about whether the guiding principles are right, and if the College should be endorsing specific measures or advocating for the routine use of outcome measures. This will be used to guide future revisions of the report.

The working group believes the proposed survey will enable it to explore the facilitators and barriers to routine outcomes data capture both locally and nationally, including:

- how to consider organisational drivers and buy in of clinical staff
- digital enablement
- understanding time points in a chronic relapsing remitting condition in the community vs. episode of therapy or hospital admission
- clinical burden/benefit and buy in
- training.

An invitation to participate in the survey will be sent to all College members and advertised via social media, at the International Congress. Analysis will be via descriptive summary of quantitative data and a thematic summary of any free text data.

**Results:**

The group intends to use the intelligence gather to inform, influence and shape policy that promotes routine outcome data capture and publish its findings for wider dissemination.

**Conclusion:**

Outcome measurement is a top priority for the RCPsych. A new Associate Registrar and working group is spearheading the College's work in this area, publishing guidance and conducting further research. Engagement and learning from our colleagues would provide critical intelligence to inform and influence future policy and strategy to enable routine outcome gathering embedded in mental health services.

